# Robot-assisted anterior resection of rectal cancer in a patient with abdominal aortic aneurysm using a monitoring laparoscope: a case report

**DOI:** 10.1097/MS9.0000000000002936

**Published:** 2025-02-11

**Authors:** Junpei Takashima, Hirotoshi Kobayashi, Ayaka Koizumi, Fumi Shigehara, Kenji Yamazaki, Daisuke Fujimoto, Fumihiko Miura, Keizo Taniguchi

**Affiliations:** Department of Surgery, Teikyo University School of Medicine, Mizonokuchi Hospital, Kawasaki, Kanagawa, Japan

**Keywords:** abdominal aortic aneurysm, contraindications, lymph node excision, rectal neoplasms, robot-assisted rectal resection, sensation

## Abstract

**Introduction and importance:**

We present the first case of robot-assisted anterior resection for rectal cancer in a patient with abdominal aortic and common iliac aneurysms using a monitoring laparoscope for enhanced safety.

**Case presentation:**

An 86-year-old man presented with bloody stool and was diagnosed with Stage IIIB rectal cancer (T3N1aM0). Preoperative computed tomography revealed a 39-mm abdominal aortic aneurysm and 25-mm left common iliac aneurysm. Robot-assisted anterior resection with D2 lymph node dissection was performed using a 5-mm laparoscope to avoid contact between robotic forceps and aneurysm. The procedure was successful, and he was discharged from the hospital on the ninth postoperative day.

**Clinical discussion:**

Robotic surgery, owing to the lack of tactile sensation, is generally contraindicated in cases of abdominal aortic aneurysms because of the risk of vascular injury. However, this case demonstrates that real-time monitoring with a 5-mm laparoscope can effectively prevent accidental vascular injury during robotic surgery for rectal cancer.

**Conclusion:**

This case illustrates the appropriate modifications by which robotic surgery for rectal cancer can be safely performed in patients with abdominal aortic or common iliac artery aneurysms.

## Introduction

Robotic surgery is widely used for rectal cancer treatment; however, its application in patients with abdominal aortic aneurysms is exceedingly rare due to the significant risks involved, and to date, there have been no reported cases of such procedures. This case highlights the successful use of robotic-assisted surgery in a patient with both rectal cancer and abdominal aortic aneurysms, emphasizing the importance of innovative approaches to safely manage such complex conditions.

This case was reported in line with the SCARE 2023 criteria[1].

## Case presentation

### Patient information and clinical findings

An 86-year-old man presented with bloody stool and history of stroke. He was on clopidogrel (75 mg once daily), and the bloody stool persisted even after discontinuation of the medication. Blood tests showed moderate anemia (hemoglobin, 8.4 g/dL).

### Diagnostic assessment

**Figure 1.**
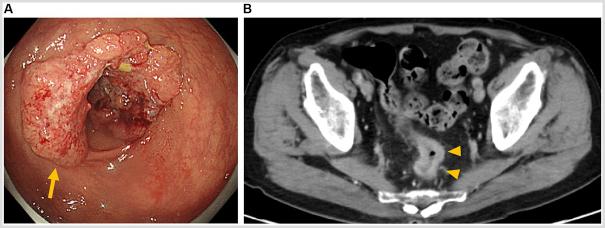
Preoperative findings. (A) Colonoscopy findings. A type 2 lesion is observed in the rectum situated 13 cm from the anal verge (arrow). (B) Abdominal contrast-enhanced computed tomography image depicting thickening of the intestinal wall with contrast enhancement in upper rectum (arrowheads).

**Figure 2.**
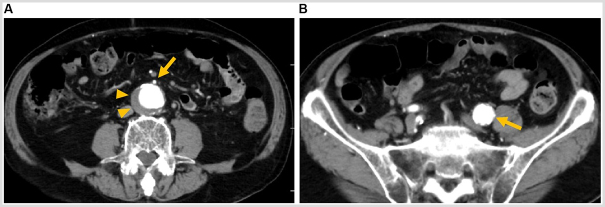
Preoperative abdominal contrast-enhanced computed tomography images. (A) A 39-mm abdominal aortic aneurysm (arrowheads) is observed caudally to the branching point of the inferior mesenteric artery (arrow). (B) A 25-mm left common iliac artery aneurysm is observed immediately cranial to the bifurcation of the internal and external iliac arteries (arrow).

Colonoscopy revealed a type 2 lesion occupying two-thirds of the rectal circumference situated 13 cm from the anal verge (Fig. 1a), which was diagnosed as moderately differentiated adenocarcinoma based on histopathologic examination. Chest and abdominal contrast-enhanced computed tomography (CT) revealed thickening of the intestinal wall with contrast enhancement in upper rectum (Fig. 1b). Enlarged paracolic lymph nodes were present without distant metastasis, leading to the preoperative diagnosis of Stage IIIB rectal cancer (T3N1aM0). A 39-mm abdominal aortic aneurysm (Fig. 2a) and a 25-mm left common iliac artery aneurysm (Fig. 2b) were incidentally discovered on CT, and neither had been previously noted. Due to the small size of the aneurysms and rectal bleeding severity, rectal cancer treatment was prioritized and robotic surgery was planned.

### Treatment and surgical intervention

**Figure 3.**
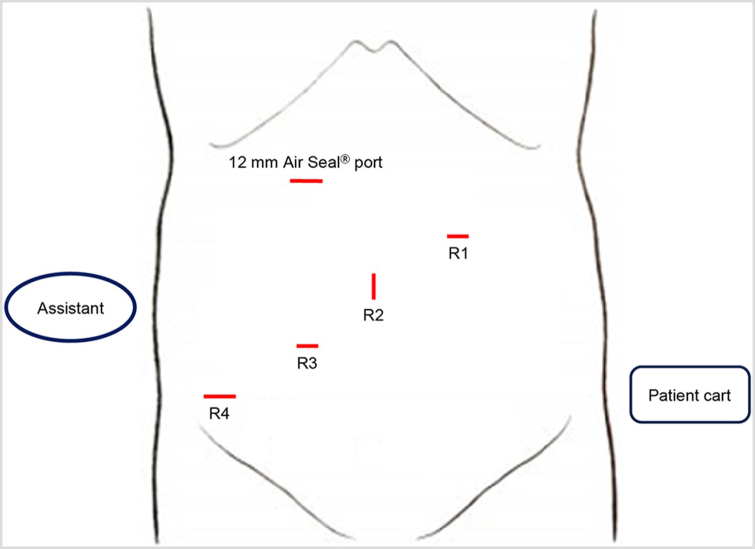
Port arrangement used in the present case. R4 is a 12-mm port, and R1–3 are 8-mm ports.

**Figure 4.**
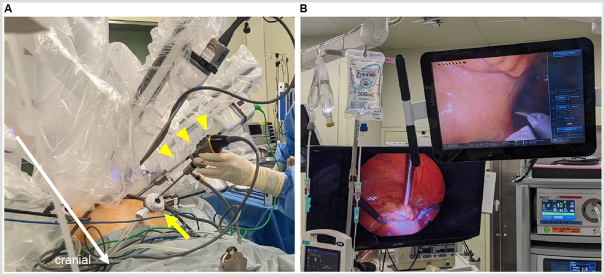
Intraoperative setup. (A) A 5-mm laparoscope (yellow arrowheads) was inserted through a 12-mm AirSeal^®^ port (yellow arrow) to observe the intraperitoneal cavity with a real-time monitor. (B) Monitors for the assistant. The upper right monitor shows the view from the robotic camera, and the lower left monitor shows the view from the 5-mm laparoscope.

Robot-assisted high anterior resection using the da Vinci Xi system (Intuitive Surgical Systems, Sunnyvale, CA, USA) was performed. The port placement is shown in Fig. 3. A 5-mm laparoscope was used in conjunction with the robotic system to prevent aneurysm rupture. A 12-mm AirSeal® (ConMed, Utica, NY, USA) port in right upper abdomen served as a port for the laparoscope assisted by another surgeon (Fig. 4a), and a 5-mm laparoscopic grasper and a 5-mm laparoscope could be simultaneously inserted through the port. Surgery was performed to ensure that the robotic instruments did not contact the aneurysms using real-time monitoring with the 5-mm laparoscope (Fig. 4b). Blood pressure was meticulously regulated to sustain a systolic measurement of 120 ± 20 mmHg.

**Figure 5.**
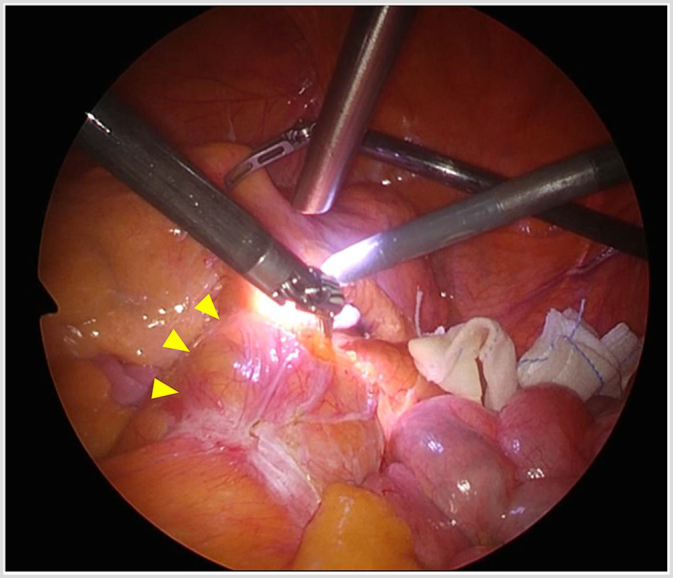
View from the 5-mm laparoscope showing abdominal aortic aneurysm (arrowhead) in close proximity during the manipulation of the inferior mesenteric artery.

**Figure 6.**
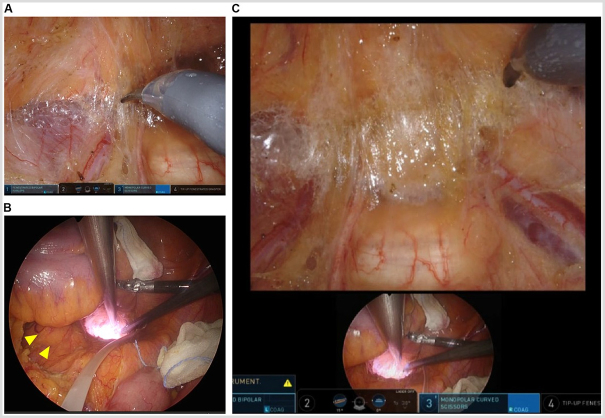
Pelvic procedure. (A) View from the robotic camera showing the dissection of the anterior layer of the pre-hypogastric nerve fascia. (B) View from the 5-mm laparoscope showing the left common iliac artery aneurysm (arrowhead). (C) TilePro system shows that surgery can proceed while simultaneously displaying the view from the 5-mm laparoscope in the surgeon’s field of view.

D2 lymph node dissection was performed (Fig. 5). After performing adequate pelvic procedures (Fig. 6a–c), the rectum was transected 2 cm from the anal side of the tumor using a SureForm® green 60-mm stapler. Anastomosis was performed using the double stapling technique, and leak test was performed to confirm the absence of anastomotic failure. The surgery lasted 3 h and 52 min, with a minimal blood loss of 5 ml.

### Follow-up and outcome

The patient’s postoperative course was uneventful, and he was discharged on postoperative day 9, without aneurysm rupture during or after surgery. Postoperative pathological findings revealed moderately differentiated adenocarcinoma with subserosal invasion without lymph node metastasis (pT3N0M0) and negative circumferential and longitudinal margins (R0). The patient did not receive postoperative adjuvant chemotherapy and was recurrence-free at 3-year follow-up.

## Discussion

To the best of our knowledge, this is the first study to report robotic surgery in patients with gastrointestinal cancer and concomitant abdominal aortic or common iliac artery aneurysm. We successfully performed robot-assisted high anterior resection of rectal cancer in a patient with abdominal aortic and common iliac artery aneurysms using a 5-mm laparoscope for real-time monitoring.

In Japan, robotic-assisted surgery for rectal cancer has been covered by insurance since April 2018, and the da Vinci Surgical System is widely used[2]. Robotic surgery allows precise manipulations due to articulated instruments, 3D visualization, tremor correction, and motion scaling. The da Vinci Xi system, which incorporates the Firefly and TilePro systems, is extensively used in clinical practice. However, a major drawback is the absence of haptic feedback, which, combined with the rigidity of the robotic arms, might lead to serious accidents. The lack of tactile sensation can result in excessive force applied to tissues, raising concerns regarding inadvertent organ damage[3]. Therefore, robotic surgery is generally not considered in patients with abdominal aortic or common iliac artery aneurysm.

Considering that this case involved Stage IIIB rectal cancer without distant metastasis, we explored the option of radiotherapy to avoid surgery due to the surgical risks associated with the abdominal aortic aneurysm. However, we ultimately determined that surgery was the most appropriate course of action. This decision was based on the patient’s history of stroke and the fact that he was on clopidogrel. The neurologist instructed that clopidogrel should be restarted and continued as soon as possible. However, the patient had to temporarily discontinue clopidogrel due to prolonged bloody stool and moderate anemia, with an Hb level of 8.4 g/dL. Surgery was chosen because resecting the rectal cancer, which was the primary source of hemorrhage, would allow for the early resumption of clopidogrel.

Abdominal aortic aneurysm is defined as the localized expansion of aorta to more than 1.5 times its normal diameter, characterized by thinning of the aortic wall^[4,5]^. The risk of rupture increases with aneurysm diameter. Overall mortality rate of patients with ruptured abdominal aortic aneurysms is high, from 65% to 85%, and 8000 and 15 000 annual deaths on average are reported in the United States and Japan, respectively[6]. The global prevalence of abdominal aortic aneurysms has been declining; however, the mortality rate at the time of rupture remains high. Therefore, the number of deaths from abdominal aortic aneurysms remains high[7]. For aneurysms over 5 cm in diameter, the risk of rupture over 5 years is as high as 25%, whereas the risk of rupture is nearly 0% for those with a diameter smaller than 5 cm. In the present case, the abdominal aortic aneurysm did not warrant treatment based on its size. Although open surgery remains the predominant treatment method for abdominal aortic aneurysm, minimally invasive approaches such as endovascular aneurysm repair and laparoscopic repair have become widely used recently. Furthermore, albeit not yet an established approach, the utility of robotic surgery has been recently suggested[8].

Blunt trauma causing abdominal aortic injury is extremely rare and often involves high-energy forces during vehicular accidents or falls from height. Rupture due to trauma occurs when intra-aortic pressure exceeds 1000–2500 mmHg[9]. Conversely, studies have reported rupture of abdominal aortic aneurysm due to low-energy blunt trauma, such as punch to the abdomen[10]. Due to their relatively large size within the abdominal cavity, vulnerability to direct forces, weakened vascular walls, and decreased elasticity and extensibility secondary to arteriosclerotic changes render abdominal aortic aneurysms at risk of rupture from considerably lower energy forces than the typical trauma-induced rupture. In robot-assisted surgery, excessive pressure might be applied to an aortic aneurysm due to the lack of tactile feedback or robotic instruments might move unexpectedly due to contact with each other in the abdominal cavity, which is often encountered by inexperienced surgeons. Thus, we predicted that robotic surgery would increase the risk of aortic aneurysm rupture compared to conventional laparoscopic surgery. To eliminate this risk, we employed a 5-mm laparoscope to continuously monitor the surgical field from a bird’s eye view, ensuring that the instruments did not contact the aneurysm, which allowed for a safe surgical procedure. As illustrated in Fig. 4b, the assistant monitored both the robotic view of the surgeon and the distant view provided by the 5-mm laparoscope using a double monitor. Furthermore, the TilePro system (Fig. 6c) allowed the surgeon to perform the surgery while simultaneously comparing the views of the laparoscopic and robotic cameras. Although contact with the abdominal aortic aneurysm was easily prevented with caution throughout the surgery, special attention was necessary during the pelvic procedure to prevent contact between the common iliac artery and the shaft of the robotic instruments. For surgeons inexperienced in robotic surgery, using a 5-mm laparoscope to observe the surgical field might be beneficial in assessing the movements of the instruments and their contact with surrounding organs.

The type of trocar used for real-time monitoring was another important consideration. In our department, a 12-mm AirSeal^®^ port is placed in the right upper abdomen as an assistant port, which is usually used to introduce a 5-mm laparoscopic grasper and a suction tube. In the present case, the same port was used to additionally insert the 5-mm laparoscope. The 12-mm AirSeal^®^ port allowed the simultaneous insertion of both instruments without any issues, enabling successful completion of surgery. Other trocars might not have allowed the implementation of this method, necessitating caution.

## Conclusions

Laparoscopic real-time monitoring is beneficial to oversee the surgical field during robotic surgery, thereby preventing accidental vascular injury. Even in patients with abdominal aortic or common iliac artery aneurysm, robotic surgery for rectal cancer is possible with appropriate modifications.

## Data Availability

Data sharing is not applicable to this article as no datasets were generated or analyzed during the current study.

## Research registration unique identifying number (UIN)

Not required, because this study is a case report.
